# Burden of extended-spectrum beta-lactamase-producing Enterobacteriaceae among cancer patients in Africa: a systematic review and meta-analysis

**DOI:** 10.1099/acmi.0.001120.v4

**Published:** 2026-06-12

**Authors:** Henry Zamarano, Benson Musinguzi, Muzafaru Twinomujuni, Catherine Khakasa, Vicent Mwesigye, Ivan Muhwezi, Edgar Mugema Mulogo, Deborah Natumanya, Simon Kawuma, Patrick Orikiriza, Jacob Stanley Iramiot

**Affiliations:** 1 Department of Microbiology, Faculty of Medicine, Mbarara University of Science and Technology, P.O Box 1410, Mbarara, Uganda; 2 Uganda Protestant Medical Bureau, P.O. Box 4127, Kampala, Uganda; 3 Department of Medical Laboratory Sciences, Faculty of Health Sciences, Muni University, P.O. Box 725, Arua, Uganda; 4 Department of Immunology and Molecular Biology, School of Biomedical Sciences, College of Health Science, Makerere University, P. O. Box 7062, Kampala, Uganda; 5 Department of Medical Laboratory Science, Faculty of Medicine, Mbarara University of Science and Technology, P.O Box 1410, Mbarara, Uganda; 6 Department of Microbiology and Immunology, Faculty of Health Sciences, Busitema University, P.O. Box 1460 Mbale, Uganda

**Keywords:** Africa, cancer, *Enterobacteriocae*, extended-spectrum beta-lactamase

## Abstract

**Background.** Extended-spectrum beta-lactamase-producing *Enterobacteriaceae* (ESBL-P*E*) exacerbate infections in cancer patients in settings where antimicrobial resistance threatens health outcomes. This study estimated the prevalence of ESBL-P*E* among cancer patients in Africa from 1 January 2010 to 31 December 2024.

**Methods.** We searched PubMed, Embase, Web of Science, CINAHL and Global Health for observational studies reporting ESBL-P*E* prevalence in cancer patients. Studies published in English from 1 January 2010 to 31 December 2024 were included. Two reviewers independently screened studies, extracted data using standardized forms and assessed the quality of articles using the Newcastle–Ottawa Scale. Pooled prevalence was calculated using a random-effects model in RStudio v4.4.2, with heterogeneity assessed with the *I*² statistic and publication bias assessed with funnel plots and Egger’s test.

**Results.** Twelve studies from 9 African countries, involving a total of 1,252 cancer patients and 643 identified events, were included. The pooled prevalence of ESBL-P*E* was 49.4% (95% CI: 36.0–62.9%, *I*²=88.3%, *P*<0.001). *Escherichia coli* and *Klebsiella pneumoniae* predominated. There was no evidence of publication bias based on funnel plot symmetry, Egger’s test (*t*=−0.35, *P*=0.73) and trim-and-fill analysis (no studies imputed; adjusted estimate unchanged). Heterogeneity was substantial (*I*²=88.3%) with a wide 95% prediction interval (22.7–76.5%). Leave-one-out sensitivity analysis confirmed estimate stability (~49%) with persistently high heterogeneity (*I*² range: 81.4–89.4%).

**Conclusions.** The high ESBL-P*E* prevalence in African cancer patients signals a critical public health issue, necessitating enhanced surveillance, antimicrobial stewardship and intentional infection control measures.

## Data availability

All data are available in the manuscript and through the supplementary file.

## Background

Antimicrobial resistance is a critical global health threat, with extended-spectrum beta-lactamase-producing *Enterobacteriaceae* (ESBL-P*E*) emerging as a significant concern, particularly for immunocompromised populations, such as cancer patients [1,4]. In Africa, where healthcare systems are often under-resourced, the burden of ESBL-P*E* is augmented by limited access to effective antibiotics, widespread antibiotic misuse and inadequate infection control measures [5,7]. Cancer patients in Africa face additional challenges, including late-stage diagnoses, limited access to specialized oncology services and high rates of comorbidities, such as human immunodeficiency virus (HIV) and diabetes, which further increase their susceptibility to multidrug-resistant infections [8,11]. Recent studies continue to report alarmingly high rates of ESBL-P*E* across various African countries, such as Egypt (60%) [12] and Ethiopia (49%) [13], highlighting the persistence of this public health crisis.

The epidemiology of ESBL-P*E* in Africa is shaped by factors such as the overuse of broad-spectrum antibiotics, poor sanitation and limited diagnostic capacity [14,15]. In oncology settings, these challenges are compounded by frequent invasive procedures, such as catheter insertion and surgeries, as well as prolonged antibiotic exposure, which elevate the risk of healthcare-associated infections [7,14, 16, 17]. The reliance on carbapenems as a last-line treatment for ESBL-P*E* infections raises concerns about the emergence of carbapenem-resistant *Enterobacteriaceae*, which severely limits therapeutic options and poses a significant public health threat [18,19]. Regional disparities in healthcare infrastructure, antibiotic prescribing practices and surveillance systems contribute to varying ESBL-P*E* prevalence across Africa, necessitating a systematic evaluation to inform intentional interventions. Previous studies in African healthcare settings have reported varying ESBL-P*E* prevalence, with higher rates in high-risk populations, such as intensive care unit patients. However, data specific to cancer patients remain limited, with most studies focusing on general hospital populations or specific pathogens [6,20,22]. Available evidence suggests that ESBL-P*E* infections in cancer patients are associated with worse clinical outcomes, including higher mortality rates and extended hospital stays [2,8, 23,26]. These findings underscore the need for a comprehensive synthesis of data to quantify the burden of ESBL-P*E* in African cancer patients, identify risk factors and characterize resistance patterns. This systematic review and meta-analysis estimated the prevalence of ESBL-P*E* among cancer patients in Africa from 1 January 2010 to 31 December 2024. By synthesizing data from observational studies, we provide a robust estimate of ESBL-P*E* prevalence, explore regional and cancer-type variations and report antimicrobial resistance patterns. Additionally, we aimed to identify gaps in the literature and propose directions for future research to strengthen infection control and antimicrobial stewardship in oncology settings.

## Methods

### Study design

This systematic review and meta-analysis were conducted in accordance with the Preferred Reporting Items for Systematic Reviews and Meta-Analyses (PRISMA) guidelines [27]. The study synthesized observational data to estimate the prevalence of ESBL-P*E* among cancer patients in Africa from 1 January 2010 to 31 December 2024.

### Study setting

The review included studies conducted in African countries, encompassing diverse healthcare settings, such as tertiary hospitals, oncology centres and community clinics.

### Search strategy

A comprehensive search was conducted in PubMed, Embase, MEDLINE, Web of Science, CINAHL and Global Health databases. Medical Subject Headings (MeSH) and keywords included ‘*Enterobacteriaceae*’, ‘Extended-Spectrum Beta-Lactamase’, ‘Cancer’, ‘Prevalence’ and ‘Africa’ (including all 54 African country names). A sample PubMed search string was (*‘Enterobacteriaceae’*[MeSH] OR ‘*Enterobacteriaceae*’) AND (‘Extended-Spectrum Beta-Lactamase’[MeSH] OR ‘ESBL’) AND (‘Cancer’[MeSH] OR ‘Neoplasm*’) AND (‘Africa’) AND (‘prevalence’ OR ‘incidence’). Searches were restricted to English-language studies published from 1 January 2010 to 31 December 2024. Searches were updated until the final analysis.

### Selection criteria

Eligible studies included observational studies (cross-sectional, cohort and case-control) or clinical trials reporting ESBL-P*E* prevalence in cancer patients (any type, adults or children) in African countries. Studies required quantitative prevalence or incidence data and specified ESBL detection methods (phenotypic or genotypic). Exclusions included case reports, case series (<10 participants), reviews, meta-analyses, editorials or studies lacking cancer patient specificity.

### Review question

The review question was ‘What is the prevalence of extended-spectrum beta-lactamase isolated from cancer patients in Africa?’

### Study selection

**Fig. 1.**
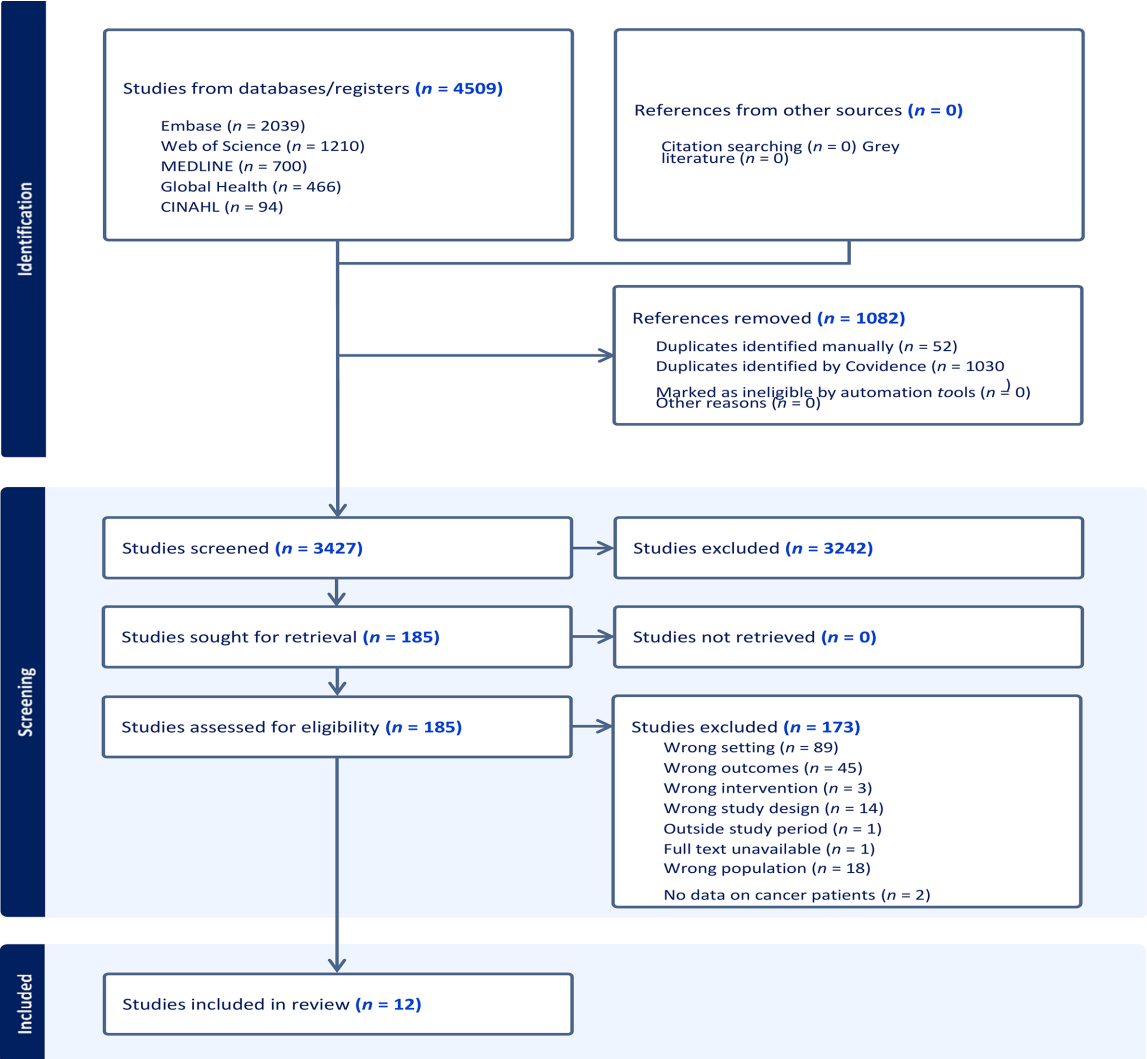
PRISMA flow diagram illustrating identification, screening, eligibility and inclusion of studies assessing ESBL-P*E* prevalence among cancer patients in Africa (1 January 2010 to 31 December 2024).

Two reviewers independently screened titles, abstracts and full texts using Covidence software. Duplicates were removed via Covidence and manual checks. Discrepancies were resolved through discussion or consultation with a third reviewer. The selection process was documented in a PRISMA flow diagram (Fig. 1), where a total of 4,509 studies were identified through different search strategies, including Embase (2039), MEDLINE (700), Web of Science (1210), Global Health databases (466) and CINAHL (94). However, no references were identified from other sources, such as citation search and grey literature.

Based on the exclusion criteria, 1,082 studies were removed, including 52 duplicates identified manually and 1,030 duplicates identified by Covidence. Further screening of the remaining 3,427 studies was done, and 3,242 studies were excluded.

Finally, 185 studies were screened, and 173 of them were excluded due to wrong setting (89), wrong outcome (85), wrong intervention [2], wrong study design [14], outside study period [1], wrong population [18] and no data [2], leaving only 12 studies that fit the inclusion criteria, as shown in Fig. 1.

### Data extraction

A standardized Microsoft Excel template captured (i) study characteristics (author, year, country, design and sample size), (ii) participant characteristics (age, gender, cancer type and comorbidities), (iii) ESBL-P*E* attributes (prevalence, species and detection methods) and (iv) antimicrobial resistance patterns. Two reviewers (H.Z. and C.K.) independently extracted data, with discrepancies resolved via consensus.

### Quality assessment

The methodological quality of the 12 studies was assessed using the Newcastle-Ottawa Scale, with overall scores ranging from 4 to 9 out of a possible nine points. Four studies demonstrated a low overall risk of bias, each achieving a score of 9; these included three cohort studies [28,30] and one retrospective study [26]. Six studies had a moderate risk of bias with scores between 6 and 7 points [2,25, 31,34]. The remaining two cross-sectional studies were assessed as having a higher risk of bias, scoring 4 [35] and 5 [17], respectively. A common area of concern contributing to lower scores, particularly in several cross-sectional studies [17,25, 34, 35], was the 'Comparability' domain, mainly due to a lack of multivariate adjustment when analysing associations. Weaknesses in the ‘Selection’ domain, such as the use of convenience sampling [31], low or unclearly reported participation rates [35] or the lack of sample size justification [34,35], also impacted the assessments.

### Data analysis

A random-effects meta-analysis was performed using RStudio v4.4.2 to calculate pooled ESBL-P*E* prevalence with 95% CIs. Heterogeneity was assessed using Cochran’s *Q* test and *I*² statistics (*I*² > 50% indicating substantial heterogeneity). Subgroup analyses explored variations by region. Publication bias was evaluated using funnel plots and Egger’s test, with trim-and-fill analysis to adjust for missing studies. Sensitivity analyses assessed the impact of study quality, inclusion criteria and missing data imputation. Results were presented in forest plots, funnel plots and tables. A qualitative narrative synthesis complemented the meta-analysis to contextualize findings.

## Results

### Characteristics of studies and participants

Of 4,509 studies identified, 12 observational studies from 9 African countries were included, of which 8 (67%) were from North Africa (Egypt, 4; Algeria, 2; Sudan, 2), 3 (25%) from East Africa (Ethiopia, Uganda, Tanzania) and 1 (8%) from West Africa (Senegal). Nine studies (75%) were cross-sectional, two (17%) retrospective and one (8%) prospective cohort. Eleven studies (92%) used phenotypic ESBL detection, such as the double-disc synergy test for the primary test, and one used automated methods. Study populations included patients with haematologic malignancies, colorectal, gastrointestinal and genitourinary cancers. Clinical samples comprised blood, urine, rectal/stool swabs, sputum, pus and wounds. *Escherichia coli* (11 studies) and *Klebsiella pneumoniae* (10 studies) were predominant, followed by *Klebsiella oxytoca*, *Proteus mirabilis*, *Enterobacter cloacae* and *Pseudomonas aeruginosa*. Comorbidities (HIV, diabetes, hypertension and neutropenia) were reported in four studies. Study characteristics are summarized in Table 1.

**Table 1.** Characteristics of included studies

Study ID	Year	Country	Region	Study design	Total* (*N*)	Events† (*n*)	Prevalence (%)	ESBL detection method	Type of cancer	Clinical sample	Bacteria identified	Comorbidities
Nurain [37]	2015	Sudan	North Africa	Cross-sectional	185	91	49.2	Double-disc synergy test	Not specified	Urine, wound pus, blood, sputum	*E. coli*, *K. pneumoniae*, *P.aeruginosa*, *Proteus* spp.	None
Temsegen [17]	2023	Ethiopia	East Africa	Cross-sectional	107	90	84.1	ChromID ESBL agar, combination disc test	Carcinoma, leukaemia, lymphoma, sarcoma, myeloma	Stool/rectal swabs	*E. coli*, *K.pneumoniae*, *K. oxytoca*, *Enterobacter cloacae*	None
Lubwama [2]	2024	Uganda	East Africa	Cross-sectional	73	54	74.0	Phenotypic testing, PCR	Haematologic cancer	Blood	*E. coli*, *K.pneumoniae*	None
El-Mahdy [34]	2015	Egypt	North Africa	Cross-sectional	75	39	52.0	Double-disc synergy test	Not specified	Sputum, urine, blood, pus, stool	*K. pneumoniae*	None
Mahmoud [25]	2020	Egypt	North Africa	Cross-sectional	110	42	38.2	Double-disc synergy test	Not specified	Urine	*E. coli*	None
Abdallah 2024 [31]	2024	Tanzania	East Africa	Cross-sectional	48	13	27.1	Double-disc synergy test (automated)	Cervix, breast, digestive, prostate, skin, blood/lymphatic	Urine	*E. coli*, *K.pneumoniae*, *Proteus mirabilis*, *Enterobacter cloacae*, *P.aeruginosa*	HIV, diabetes, hypertension, asthma
Zain [33]	2022	Sudan	North Africa	Cross-sectional	44	15	34.1	Double-disc synergy test	Not specified	Blood	*E. coli*, *K. pneumoniae*, *P.aeruginosa*, *C. freundii*	None
Touati [35]	2020	Algeria	North Africa	Prospective cohort	42	6	14.3	Chromogenic media	Colorectal cancer	Rectal swabs	*E. coli, K.pneumoniae*	Diabetes, hypertension
Medboua-Benbalagh [28]	2017	Algeria	North Africa	Cross-sectional	171	93	54.4	Double-disc synergy test	Haematologic (leukaemia, lymphoma), neuroblastoma, nephroblastoma, sarcomas	Rectal swabs	*E. coli, K.pneumoniae*, *K. oxytoca*, *Enterobacter cloacae*, *C. freundii*	None
Youssef [30]	2020	Egypt	North Africa	Retrospective	62	28	45.2	MALDI-TOF Vitek MS, Vitek 2	Haematologic malignancies, lymphomas, solid tumours	Blood	*E. coli*, *K. pneumoniae*, *Enterobacter cloacae*, *P. aeruginosa,* *S. marcescens*	None
Mohamed [26]	2023	Egypt	North Africa	Retrospective	319	162	50.8	Double-disc synergy test	Gastrointestinal, genitourinary, pancreatic tumours	Urine, respiratory, blood	*E. coli*, *K.pneumoniae*	Neutropenia
Ndir [29]	2016	Senegal	West Africa	Retrospective	16	10	62.5	Disc diffusion synergy method	Not specified	Blood, urine, surgical sites	*E. coli*, *K. pneumoniae*, *K. oxytoca*, *Proteus mirabilis*, *Enterobacter cloacae*	Meningitis

*Total number of cancer patients in the study.

†Number of patients with ESBL-P*E* in the study.

### Prevalence of ESBL-P*E*

**Fig. 2.**
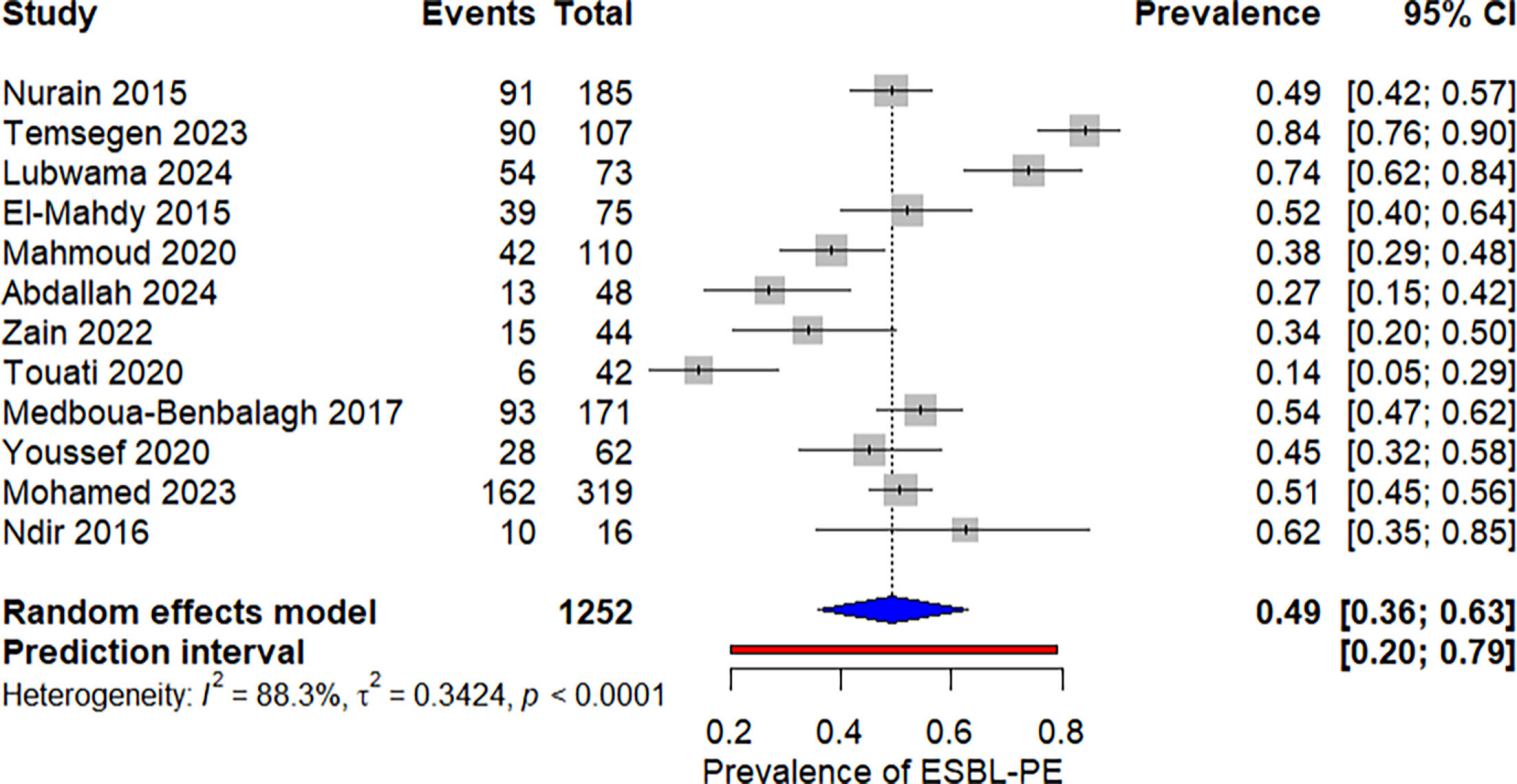
Forest plot showing pooled prevalence of ESBL-P*E* among cancer patients in Africa.

**Fig. 3.**
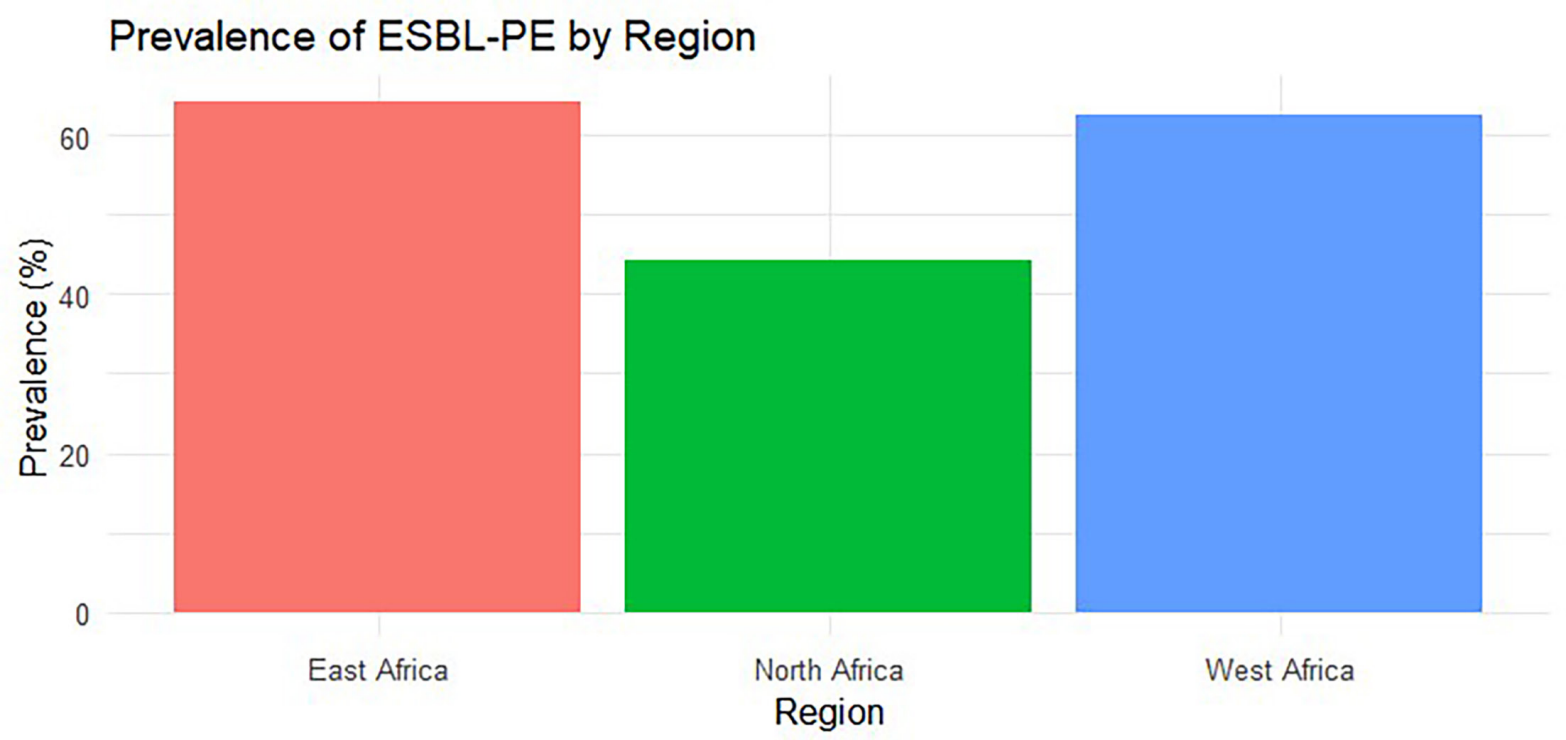
Graph showing prevalence of ESBL-P*E* among cancer patients by regions.

The pooled prevalence of ESBL-P*E* was 49.4% (95% CI: 36.0–62.9%, *I*²=88.3%, *P*<0.001) (Fig. 2). Subgroup analyses revealed higher prevalence in East Africa (64.2%, 95% CI: 5.4–98.3%, *I*²=95.2%, *P*<0.0001) than North Africa (44.3%, 95% CI: 34.0–55.2%, *I*²=74.2%, *P*=0.0003). Only one study was reported from West Africa with a prevalence of 62.5% (Fig. 3).

### Antimicrobial resistance patterns

There was a reported high resistance to cephalosporins (*E. coli*: median 89.5%, range 55.6–100%; *K. pneumoniae:* 90%, 74.5–100%) and trimethoprim-sulfamethoxazole (*E. coli*: 87%, 71–98%; *K. pneumoniae:* 90%, 71–95%). Fluoroquinolone resistance was higher in East Africa (median: 90%) than in North Africa (55%). Carbapenem resistance was low in *E. coli* (median: 11.1%, range 0–30%) but moderate in *K. pneumoniae* (median: 70%, range 0–70%), with high sensitivity (*E. coli*: 88.9%, 70–100%). Resistance patterns were consistent across patient- and sample-level studies (Table 2).

**Table 2.** Antimicrobial resistance and sensitivity patterns

Antibiotic class	Bacteria	Median resistance (%)	Range resistance (%)	Studies reporting resistance	Median sensitive (%)	Range sensitive (%)	Studies reporting sensitivity
Cephalosporins	*E. coli*	89.5	55.6–100	Abdallah (55.6%) [31], Lubwama (89%) [2], Mahmoud (81.8%) [25], Mohamed (90%) [26], Zain (89.5%) [33], Medboua-Benbalagh [28]	10	0–44.4	Abdallah (44.4%) [31], Mohamed (10%) [26], Touati (0%) [35], Medboua-Benbalagh (0%) [28]
Cephalosporins	*K. pneumoniae*	90	74.5–100	El-Mahdy (96%) [34], Lubwama (84%) [2], Mahmoud (74.5%) [25], Mohamed (90%) [26], Medboua-Benbalagh (100%) [28]	10	4–20	El-Mahdy (4%) [34], Mohamed (10%) [26], Medboua-Benbalagh (20%) [28]
Carbapenems	*E. coli*	11.1	0–30	Abdallah (11.1%) [31], Lubwama (0%) [2], Mohamed (30%) [26], Touati (0%) [35], Medboua-Benbalagh (0%) [28]	88.9	70–100	Abdallah (88.9%) [31], Lubwama (78%) [2], Mohamed (70%) [26], Touati (100%) [35], Medboua-Benbalagh (100%) [28]
Carbapenems	*K. pneumoniae*	4	0–100	El-Mahdy (4%) [34], Lubwama (0%) [2], Mohamed (70%) [26], Touati (0%) [35], Medboua-Benbalagh (100%) [28]	30	30–100	El-Mahdy (96%) [34], Lubwama (84%) [2], Mohamed (30%) [26], Touati (100%) [35], Medboua-Benbalagh (0%) [28]
Aminoglycosides	*E. coli*	41.7	25–71	Abdallah (41.7%) [31], Lubwama (65%) [2], Mahmoud (42.73%) [25], Mohamed (25%) [26], Touati (0%) [35], Medboua-Benbalagh (71%) [28]	58.3	25–78	Abdallah (58.3%) [31], Lubwama (78%) [2], Mohamed (75%) [26], Touati (0%) [35], Medboua-Benbalagh (29%) [28]
Aminoglycosides	*K. pneumoniae*	60	50–90	El-Mahdy (90%) [34], Lubwama (0%) [2], Mohamed (60%) [26], Touati (0%) [35], Medboua-Benbalagh (50%) [28]	50	50–61	El-Mahdy (75%) [34], Mohamed (50%) [26], Touati (0%) [35], Medboua-Benbalagh (61%) [28]
Fluoroquinolones	*E. coli*	60	44.4–90	Abdallah (44.4%) [31], Lubwama (90%) [2], Mahmoud (60%) [25], Mohamed (90%) [26], Touati (33.3%) [35], Zain (23%) [33], Medboua-Benbalagh (55%) [28]	44.4	10–45	Abdallah (44.4%) [31], Mohamed (10%) [26], Touati (0%) [35], Medboua-Benbalagh (45%) [28]
Fluoroquinolones	*K. pneumoniae*	79	27–90	El-Mahdy (79%) [34], Lubwama (75%) [2], Mahmoud (45.45%) [25], Mohamed (90%) [26], Medboua-Benbalagh (27%) [28]	21	10–73	El-Mahdy (21%) [34], Mohamed (10%) [26], Medboua-Benbalagh (73%) [28]
Trimethoprim-sulfamethoxazole	*E. coli*	87	71–98	Abdallah (88.9%) [31], El-Mahdy (71%) [34], Lubwama (98%) [2], Mahmoud (80%) [25], Mohamed (95%) [26], Medboua-Benbalagh (87%) [28]	na	na	na
Trimethoprim-sulfamethoxazole	*K. pneumoniae*	90	71–95	El-Mahdy (71%) [34], Lubwama (88%) [2], Mahmoud (80%) [25], Mohamed (95%) [26], Medboua-Benbalagh (90%) [28]	na	na	na
Colistin	*E. coli*	0	0–0	Mahmoud (32.73%) [25], Mohamed (0%) [26]	100	100–100	Mahmoud (67.27%) [25], Mohamed (100%) [26]
Colistin	*K. pneumoniae*	6	6–6	Mohamed (6%) [26]	94	94–94	Mohamed (94%) [26]

na indicates no studies reported numerical sensitivity data.

### Publication bias and sensitivity analysis

**Fig. 4.**
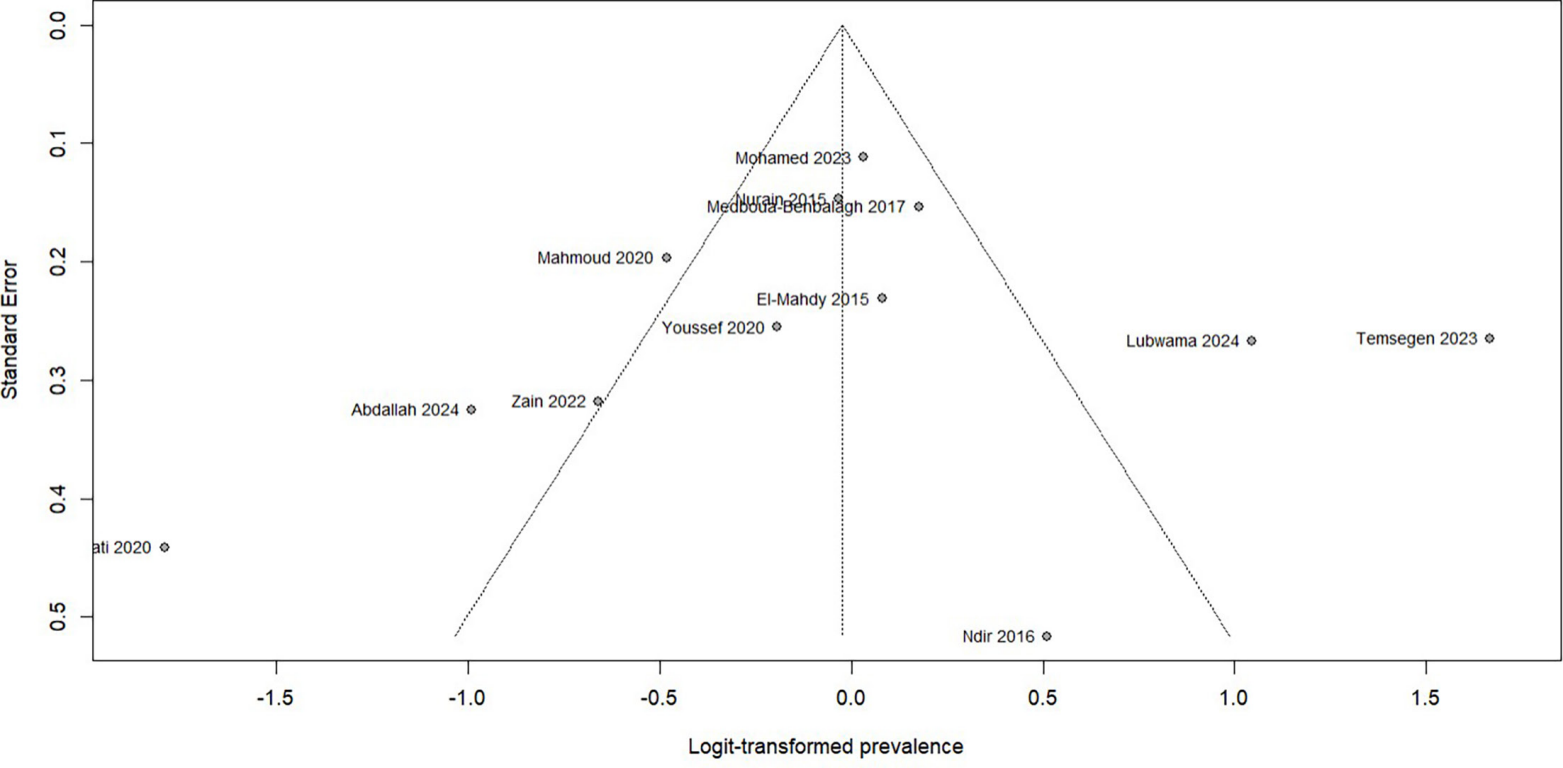
Funnel plot of pooled prevalence among cancer patients.

The funnel plot showed an approximately symmetrical distribution of studies around the pooled logit-transformed prevalence, with greater dispersion among less precise estimates, indicating no apparent evidence of publication bias or small-study effects (Fig. 4).

### Egger’s test for publication bias

Egger’s regression test showed no significant evidence of funnel plot asymmetry (*t*=−0.35, df=10, *P*=0.73) (*P*>0.05), indicating no publication bias. This is consistent with the trim-and-fill analysis, which did not impute any missing studies.

### Trim-and-fill analysis

This did not impute any missing studies, indicating no evidence of publication bias. The adjusted pooled prevalence of ESBL-P*E* among cancer patients remained 49.4% (95% CI: 40.4–58.5%), consistent with the original estimate (49.4%, 95% CI: 36.0–62.9%). Heterogeneity remained high (*I*²=88.3%), and the 95% prediction interval (22.7–76.5%) reflects substantial variability across studies.

### Sensitivity analysis

The leave-one-out sensitivity analysis demonstrated the robustness of the pooled prevalence estimate, with no single study exerting a substantial influence on the overall result (Table 3).

**Table 3.** Sensitivity analysis using the leave-one-out approach. Sensitivity analysis using a leave-one-out approach showed that the pooled prevalence of ESBL-P*E* among cancer patients remained stable (≈49%) regardless of which study was omitted. Heterogeneity also remained high (*I*² range: 81.4–89.4%), indicating that no single study unduly influenced the overall estimate

Study removed	Pooled prevalence (%)	95% CI lower	95% CI upper	Tau²	*I*² (%)
Nurain [37]	49.3	39	59.7	0.426	89.3
Temsegen [17]	46	38.3	53.5	0.194	81.4
Lubwama [2]	47.1	38.3	60.3	0.304	87.3
El-Mahdy [34]	49.1	39.8	60.1	0.378	89.4
Mahmoud [25]	50.5	42.3	59.8	0.356	88.4
Abdallah [31]	51.4	42.4	60.6	0.318	88
Zain [33]	50.8	42	59.9	0.342	88.8
Touati [35]	52.4	43.8	61.7	0.281	86.9
Medboua-Benbalagh [28]	48.8	38.8	55.5	0.415	89.2
Youssef [30]	49.2	39.9	58.9	0.369	89.3
Mohamed [26]	49.1	38.6	60.1	0.475	89.4
Ndir [29]	48.7	39.2	57.9	0.349	89.3

## Discussion

This review study aimed to estimate the prevalence and characterize resistance patterns of ESBL-P*E* among cancer patients in Africa from 1 January 2010 to 31 December 2024. In our analysis of 12 studies across nine African countries, we found a high pooled prevalence of ESBL-P*E* (49.4%). This figure significantly exceeds global estimates in general hospital populations, where they reported a pooled prevalence of ESBL-P*E* colonization of 19% (95% CI: 8–32). When stratified by region, Asia had a pooled prevalence of 31% (95% CI: 4–69%) and Europe at 15% (95% CI: 10–21%) [36], and this shows the heightened vulnerability of cancer patients in resource-constrained African settings, where infection control and antimicrobial stewardship may be wanting [2,5, 7].

Our findings are consistent with prior reports highlighting the unbalanced burden of antimicrobial resistance among immunocompromised populations in Africa [6,8]. Notably, East Africa exhibited the highest regional prevalence (64.2%), compared to 44.3% in North Africa. This may reflect regional incongruences in healthcare infrastructure, laboratory capacity and empirical prescribing practices. Frequent empirical use of broad-spectrum antibiotics, inadequate microbiological diagnostics and inconsistent infection prevention and control protocols likely contribute to these differences [5,7, 14].

*E. coli* and *K. pneumoniae* were the most commonly isolated species across all included studies, consistent with global trends [6,25, 26]. These pathogens displayed extensive resistance to cephalosporins and fluoroquinolones, with preserved carbapenem susceptibility in most studies. However, *K. pneumoniae* showed moderate carbapenem resistance in some settings, raising concerns and affirming the emergence of carbapenem-resistant *Enterobacteriaceae*, particularly in countries lacking access to newer antibiotics or robust stewardship programmes [18,19]. This trend poses serious implications for treatment outcomes in oncology patients, where delays in appropriate therapy can be fatal [23].

We observed a wide range of ESBL-P*E* prevalence across studies (12–84.1%), attributable to variability in diagnostic methods, cancer types, clinical samples and study designs. While most studies relied on phenotypic methods like the double-disc synergy test as a primary identification test, only one incorporated molecular testing, which may limit sensitivity and species-level identification [2,31]. Furthermore, studies varied in the type of cancers included, ranging from haematologic malignancies to solid tumours, each with distinct risk profiles for infection usually attributable to differences in immunosuppression, catheter use and chemotherapy intensity [2,17, 26].

Importantly, only a few studies reported patient comorbidities or clinical outcomes, limiting the capacity to analyse risk factors for ESBL-P*E* acquisition or its direct impact on morbidity and mortality. However, the studies that did report outcomes consistently linked ESBL-P*E* colonization or infection with longer hospital stays, increased healthcare costs and higher mortality, mirroring global findings [23,26,28].

The high prevalence of ESBL-P*E* found in rectal swabs, urine and blood highlights the colonization–infection continuum and the importance of routine surveillance in high-risk populations. Particularly concerning is the detection of ESBL-P*E* in faecal samples from outpatients with cancer, suggesting possible community transmission, which has been increasingly documented in Africa [13,14, 17]. This calls for integration of ESBL-P*E* screening into oncology care, especially prior to initiating chemotherapy or stem cell transplantation [30].

Although carbapenem resistance was observed in some studies, particularly among *K. pneumoniae*, most included studies did not investigate underlying resistance mechanisms such as carbapenemase production, porin loss or efflux pump overexpression. Therefore, it was not possible to definitively attribute carbapenem resistance to carbapenemase activity alone. In several settings, reduced carbapenem susceptibility may reflect the combined effect of ESBL production with altered membrane permeability or efflux mechanisms, as previously described in *Enterobacterales*. This represents an important knowledge gap and underscores the need for molecular surveillance in African oncology settings.

## Limitations of the study

The representation of ESBL-P*E* detection was inconsistent across studies, where some studies employed phenotypic rather than molecular methods for ESBL detection, which could impact the accuracy of the results. The meta-analysis also revealed high statistical heterogeneity (*I*² range: 81.4–89.4%), likely attributable to differences in diagnostic approaches, study populations and reporting practices; although sensitivity analyses and trim-and-fill procedures were conducted to address this, residual heterogeneity persisted. Furthermore, the generalizability of the findings is limited by the lack of data from Central and Southern Africa. The relatively small number of studies [12] included in the meta-analysis could also affect the precision of the estimated pooled prevalence of ESBL-P*E* among cancer patients in Africa, which was found to be 49.4%. There existed variability in reporting across included studies. Some studies reported ESBL-P*E* prevalence at the patient level, while others reported isolate-level data, potentially affecting the comparability of prevalence estimates. These limitations may have led to overestimation or underestimation of the true ESBL-P*E* burden and contribute to the observed heterogeneity. Therefore, findings should be interpreted with caution, particularly when extrapolating to underrepresented African regions.

## Conclusions

The high prevalence of ESBL-P*E* among cancer patients in Africa highlights a critical public health threat. Strengthened surveillance, antimicrobial stewardship and access to diagnostic tools are urgently needed. Targeted interventions and region-specific policies are essential to curb resistance, improve patient outcomes and protect vulnerable oncology populations across the continent.

## Supplementary material

10.1099/acmi.0.001120.v4 Supplementary Material 1.

## Abbreviations

ESBL-P*E*: Extended-spectrum beta-lactamase-producing *Enterobacteriaceae*
HIV: human immunodeficiency virus
MeSH: Medical Subject Headings
PRISMA: Preferred Reporting Items for Systematic Reviews and Meta-Analyses
